# Preferential Enhancement of Sensory and Motor Axon Regeneration by Combining Extracellular Matrix Components with Neurotrophic Factors

**DOI:** 10.3390/ijms18010065

**Published:** 2016-12-29

**Authors:** Daniel Santos, Francisco González-Pérez, Guido Giudetti, Silvestro Micera, Esther Udina, Jaume Del Valle, Xavier Navarro

**Affiliations:** 1 Institute of Neurosciences and Department of Cell Biology, Physiology and Immunology, Universitat Autònoma de Barcelona, E-08193 Bellaterra, Spain; dani.santos.1988@hotmail.com (D.S.); francisco.gzlez@gmail.com (F.G.-P.); esther.udina@uab.cat (E.U.); jaume.delvalle@uab.cat (J.D.V.); 2 Centro de Investigación Biomédica en Red sobre Enfermedades Neurodegenerativas (CIBERNED), E-08193 Bellaterra, Spain; 3 The BioRobotics Institute, Scuola Superiore Sant’Anna, Viale Rinaldo Piaggio 34, 56025 Pontedera, Italy; g.giudetti@sssup.it (G.G.); micera@sssup.it (S.M.); 4 Bertarelli Foundation Chair in Translational NeuroEngineering, Translational Neural Engineering Laboratory, Center for Neuroprosthetics and Interfaculty Institute of Bioengineering, School of Engineering, Ecole Polytechnique Federale de Lausanne (EPFL), CH-1015 Lausanne, Switzerland

**Keywords:** neurotrophic factors, BDNF, NGF, NT3, extracellular matrix, motor axons, sensory axons, nerve regeneration, reinnervation

## Abstract

After peripheral nerve injury, motor and sensory axons are able to regenerate but inaccuracy of target reinnervation leads to poor functional recovery. Extracellular matrix (ECM) components and neurotrophic factors (NTFs) exert their effect on different neuronal populations creating a suitable environment to promote axonal growth. Here, we assessed in vitro and in vivo the selective effects of combining different ECM components with NTFs on motor and sensory axons regeneration and target reinnervation. Organotypic cultures with collagen, laminin and nerve growth factor (NGF)/neurotrophin-3 (NT3) or collagen, fibronectin and brain-derived neurotrophic factor (BDNF) selectively enhanced sensory neurite outgrowth of DRG neurons and motor neurite outgrowth from spinal cord slices respectively. For in vivo studies, the rat sciatic nerve was transected and repaired with a silicone tube filled with a collagen and laminin matrix with NGF/NT3 encapsulated in poly(lactic-co-glycolic acid) (PLGA) microspheres (MP) (LM + MP.NGF/NT3), or a collagen and fibronectin matrix with BDNF in PLGA MPs (FN + MP.BDNF). Retrograde labeling and functional tests showed that LM + MP.NGF/NT3 increased the number of regenerated sensory neurons and improved sensory functional recovery, whereas FN + MP.BDNF preferentially increased regenerated motoneurons and enhanced motor functional recovery. Therefore, combination of ECM molecules with NTFs may be a good approach to selectively enhance motor and sensory axons regeneration and promote appropriate target reinnervation.

## 1. Introduction

After peripheral nerve injury, transected axons in the distal stump are disconnected from the neuronal body and undergo Wallerian degeneration, thus leading to denervation of peripheral organs [1]. Axotomized neurons switch to a growth state, and non-neuronal cells in the distal stump undergo activation and dedifferentiation to sustain nerve regeneration [2,3]. Even though axons are able to regenerate after nerve transection, axons grow randomly among the endoneurial tubules in the distal nerve, so that the accuracy of target reinnervation is usually poor, resulting in limited functional recovery [4,5,6,7]. Therefore, a pro-regenerative environment that selectively guides motor and sensory axons to regenerate into different branches of the injured nerve may be useful to increase the options of specific target reinnervation. Selective regeneration of different axonal populations would be also useful in the field of neuroprosthetics, as separating motor axons from sensory axons in mixed nerves will functionally improve selective recording and stimulation for providing bidirectional communication with the prosthesis [8,9].

After peripheral nerve injury, the generation of a pro-regenerative environment involves the upregulation and secretion of extracellular matrix (ECM) components, such as collagen type IV, laminin and fibronectin, and the secretion of different neurotrophic factors (NTFs), such as nerve growth factor (NGF), brain-derived neurotrophic factor (BDNF), neurotrophin-3 (NT3), and glial derived neurotrophic factor (GDNF), among others [10]. ECM components interact with integrin heterodimer receptors that are highly expressed in growth cones and non-neuronal cells, both during development and after injury [11,12], promoting axonal guidance, cell adhesion and migration. For instance, laminin substrates enhance elongation of sensory neurites in vitro when compared to collagen or fibronectin containing scaffolds, whereas fibronectin substrates promote neurite elongation of motor neurons from SC slices in vitro [13,14]. On the other hand, the interaction between NTFs and their different receptors, such as Trk/p75 receptors or GDNFR/RET receptors, promotes survival and axonal regeneration of different neuronal populations. For example, we found that NGF selectively promotes sensory neurite outgrowth, whereas BDNF or fibroblast growth factor (FGF) preferentially increase motor neurite outgrowth in vitro [15].

Several reports have demonstrated that the pattern of NTFs expressed in denervated Schwann cells after nerve injury is different between motor and sensory nerve branches, and that motor and sensory axons also express different cell adhesion molecules that may promote differential binding to ECM molecules [16,17,18,19]. Manipulation of some of these differential biomarkers may play a role in promoting selective regeneration of different axonal populations and improving subsequent accurate reinnervation. However, the majority of the studies have only investigated the effect of these molecules individually, disregarding the synergistic interactions of ECM molecules with NTFs [20].

Therefore, in this study, we tested whether the combination of different NTFs and ECM components, that were previously shown to produce a selective although limited stimulus for either sensory or motor axons regeneration [14,21], was able to produce a synergistic and selective pro-regenerative effect on motor and sensory neurons. Briefly, addition of both LM and NGF/NT3 or FN and BDNF increased the amount of sensory and motor neurite outgrowth, respectively, in culture models. Moreover, these effects were maintained in vivo in adult animals as sensory and motor axonal regeneration as well as functional recovery was enhanced after treating nerve injuries with a nerve conduit prefilled with the same combinations of NTFs and ECM components.

## 2. Results

### 2.1. In Vitro Effects of Combining Neurotrophic Factors (NTFs) and Extracellular Matrix (ECM) Substrates on Neurite Outgrowth

**Figure 1:**
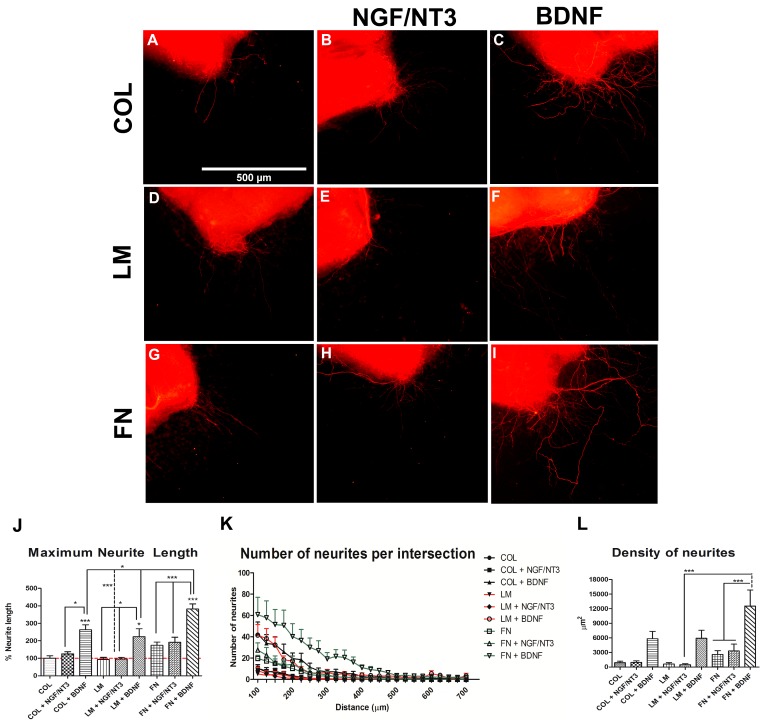
(**A**–**I**) RT97 stained neurites from spinal cord slices cultured within a 3D collagen matrix alone, and with addition of NGF/NT3 or brain-derived neurotrophic factor (BDNF) (**A**–**C**); with 20% laminin, plus NGF/NT3 or BDNF (**D**–**F**); and with 20% fibronectin, plus NGF/NT3 or BDNF (**G**–**I**); plots representing maximum neurite length in the different DRG culture conditions (**J**); quantification of the number of neurites grown at increasing distance from the DRG body, dashed line represents the values for COL control group (100%) (**K**); and plots of the quantified area under each curve of K graph (**L**). Data expressed as mean ± SEM. * *p* < 0.05 and *** *p* < 0.001.

To study the effect of different combinations of NTFs and ECM components on motor and sensory neurons outgrowth, we first performed an in vitro screening using spinal cord (SC) slices and DRG explants. In cultures of SC slices (Figure 1), addition of FN to the matrix doubled the maximum neurite length whereas addition of LM did not increase it compared to the control COL matrix (Figure 1J).

Addition of BDNF but not of NGF/NT3 to the different substrates enhanced neurite length. Furthermore, the combination of FN + BDNF significantly enhanced maximum neurite length and density of neurites compared to the other single and combined groups. Thus, while groups with either FN or BDNF present an increase in the amount and length of neurites, the combination of the two factors shows a synergistic effect with significant differences with respect to all the other groups. On the other hand, LM or NGF/NT3 alone or combined did not show any improvement (Figure 1J–L).

**Figure 2:**
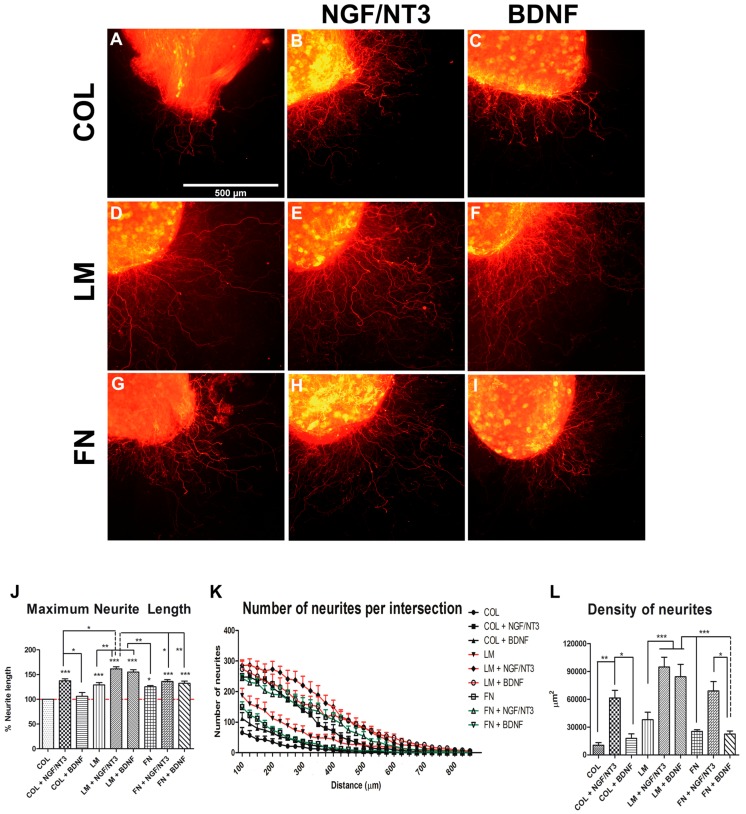
(**A**–**I**) RT97 stained neurites from DRG neurons cultured within a 3D collagen matrix alone, and with addition of NGF/NT3 or BDNF (**A**–**C**); with 20% laminin, plus NGF/NT3 or BDNF (**D**–**F**); and with 20% fibronectin, plus NGF/NT3 or BDNF (**G**–**I**); (**J**) plots representing maximum neurite length in the different culture conditions, dashed line represents the values for COL control group (100%); (**K**) quantification of the number of neurites grown at increasing distance from the cord slice; and (**L**) plots of the quantified area under each curve of K graph. Data expressed as mean ± SEM. * *p* < 0.05, ** *p* < 0.01 and *** *p* < 0.001.

In DRG explants (Figure 2), addition of LM or FN into the collagen matrix similarly enhanced the maximum neurite length (Figure 2J) compared to collagen alone.

The addition of NGF/NT3 or BDNF into COL, LM and FN substrates enhanced maximum neurite length compared with COL substrate except COL + BDNF cultures. It is noteworthy that only LM + NGF/NT3 group showed differences in maximum neurite length with respect to all the other culture conditions except with LM + BDNF. The addition of NGF and NT3 enhanced significantly the density of neurites in all the substrates, being LM + NGF/NT3 the most prominent. In contrast, addition of BDNF only promoted neurite growth with LM but not when combined with COL or FN (Figure 2L).

These results indicate that combination of FN + BDNF shows synergistic effects on motor neurons promoting neurite elongation and arborization, with weaker effect on sensory elongation, whereas LM + NGF/NT3 is the only condition that exhibits a synergistic effects on sensory but not on motor neurons based on the enhancement observed in neurite elongation and arborization.

### 2.2. In Vivo Effects of Combination of NTF and ECM Substrates on Nerve Regeneration

**Figure 3:**
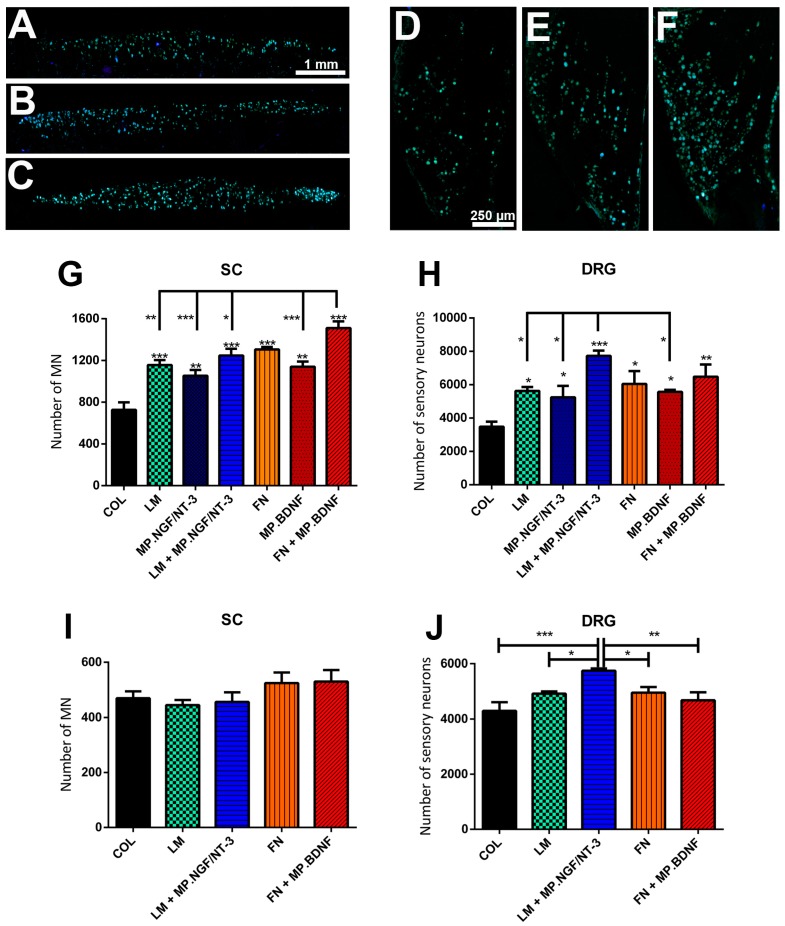
(**A**–**F**) Representative micrographs of neurons retrolabeled with FG in the spinal cord (**A**–**C**); and DRG (**D**–**F**) of rats after sciatic nerve section and repair with a nerve conduit filled: with COL (**A**,**D**); LM + MP.NGF/NT3 (**B**,**E**); or FN + MP.BDNF (**C**,**F**). (**G**–**H**) Histogram of the number of regenerated motor neurons in the spinal cord (**G**) and sensory neurons in the DRG (**H**) in the short term (20 days after injury) study. (**I**,**J**) Histogram of the number of regenerated motor neurons in the spinal cord (**I**) and sensory neurons in the DRG (**J**) after application of FG retrotracer at the ankle level 75 days after injury. Data expressed as mean ± SEM. * *p* < 0.05, ** *p* < 0.01 and *** *p* < 0.001.

We performed first an in vivo study in which the sciatic nerve was transected and repaired with silicone tubes filled with matrices composed of COL, COL + MP.NGF/NT3, LM, LM + MP.NGF/NT3, COL + MP.BDNF, and FN and FN + MP.BDNF to elucidate if the conditions with a more relevant effect in vitro had a similar effect in vivo. The three NTFs (NGF, NT3 and BDNF) were encapsulated in PLGA microspheres to allow a sustained release during the time that axons regenerate across the tube [21]. All rats showed evidence of axonal regeneration, as judged by the retrograde labeling of motor and sensory neurons with Fluorogold (FG) (Figure 3A–F).

Regarding the number of regenerated motor neurons, all groups with addition of ECM and NTFs showed significant differences with respect to the COL group (*p* < 0.001, Figure 3G), being the FN + MP.BDNF group the one with the highest effect, that was also significantly higher compared to all other groups except with FN. For sensory neurons, the group LM + MP.NGF/NT3 showed the highest number of regenerated neurons, and all other groups had better results than the COL control (Figure 3H).

We then tested whether this preferential effect is maintained in a long term study. For this purpose, we compared groups of rats similar to the short term study but leaving an 8 mm gap between stumps, since this more challenging gap allows to better elucidate differences between groups [22], and applying the FG retrotracer to the tibial nerve at the ankle at 75 dpi. In this case, no significant differences in the number of regenerated motor neurons were observed between groups (Figure 3I). However, more regenerated sensory neurons were counted in the LM + MP.NGF/NT3 group compared to the other four groups (Figure 3J).

Despite the preferential effects observed in vitro appear less marked in vivo, these results indicate that FN + MP.BDNF mainly favors motor axon regeneration, whereas LM + MP.NGF/NT3 is more effective to promote sensory axon regeneration.

### 2.3. Combination of FN and BDNF Promotes Motor Functional Recovery at Long Term

**Figure 4:**
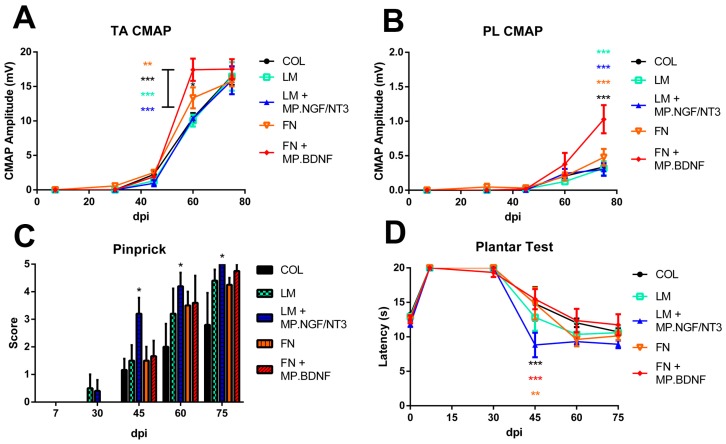

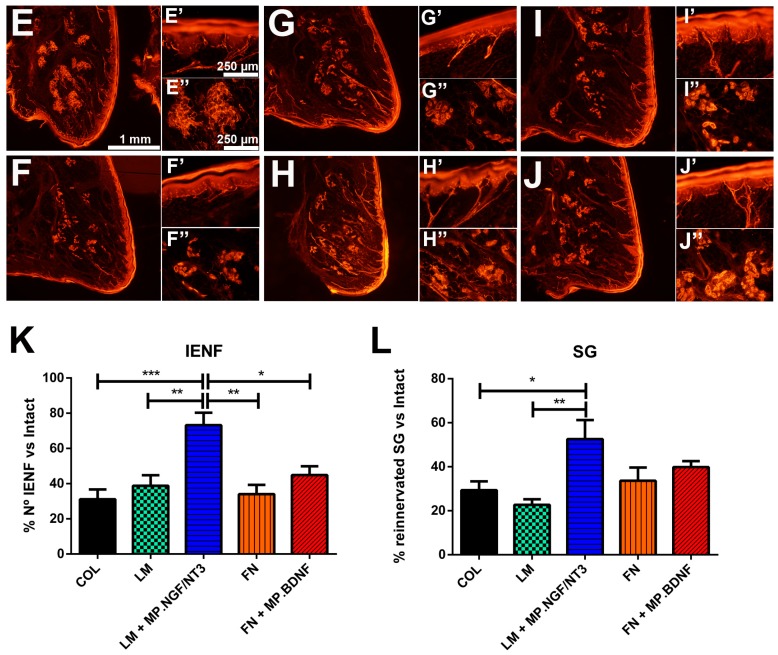
FN + MP.BDNF and LM + MP.NGF/NT3 enhance motor and sensory functional recovery, respectively. (**A**,**B**) Mean amplitude of the CMAP in TA (**A**) and PL (**B**) muscles during follow-up. ** *p* < 0.01 and *** *p* < 0.001 FN + MP.BDNF vs. all other groups referenced by the color of the star. (**C**) Pinprick score in the different groups during follow-up; * *p* < 0.05. (**D**) Latency of withdrawal response to thermal stimuli in the lateral part of the paw during follow-up; ** *p* < 0.01 and *** *p* < 0.001 LM + MP.NGF/NT3 vs. all other groups referenced by the color of the star. (**E**–**J**) Representative images of plantar pads immunolabeled against PGP in: an intact rat (**E**); COL (**F**); LM (**G**); LM + MP.NGF/NT3 (**H**); FN (**I**); and FN + MP.BDNF (**J**) treated animals. Insets: Detail of axons stained with PGP innervating epidermis and SGs. (**K**,**L**) Percentage of reinnervated IENF (**K**) and reinnervated SGs (**L**) vs. intact values. * *p* < 0.05, ** *p* <0.01 and *** *p* < 0.001. Data is presented as mean ± SEM.

We tested if the effects seen on regeneration in the short and long term studies had impact on muscle reinnervation and functional recovery. Nerve conduction tests provided first evidence of reinnervation of the TA muscle at 45 dpi in all the groups (Figure 4A). The amplitude of the CMAP increased during the follow-up. At 60 dpi, the FN + MP.BDNF group showed higher amplitude than all the other groups (*p* < 0.01). FN group also showed significant differences with respect to COL group (*p* < 0.05). However, all groups reached similar CMAP amplitude at 75 dpi. In the more distal plantar muscles, reinnervation started later compared to TA muscle. In this case, the CMAP amplitude at 75 dpi was significantly higher in FN + MP.BDNF group compared to all the other groups (*p* < 0.001, Figure 4B). No differences in the CMAP latency were observed between groups.

These results suggest that an intratubular matrix containing FN and BDNF promotes motor axon regeneration and reinnervation of target muscles, whereas LM and LM + NGF/NT3 groups showed values similar to the COL control group. The faster muscle reinnervation found in group FN + MP.BDNF is of relevance considering the longer distance that has to be regenerated in injured human nerves.

### 2.4. Combination of LM and NGF/NT3 Promotes Sensory Functional Recovery at Long Term

In parallel, we assessed sensory functional recovery to mechanical and thermal stimuli in the hind paw. For the pinprick test, LM and LM + MP.NGF/NT3 groups presented the first positive response at 30 dpi, whereas no response was observed in the other groups at this time point. At later time points, all groups showed positive responses, being LM + MP.NGF/NT3 the only group with significantly higher scores compared to the control group (*p* < 0.05; Figure 4C).

Withdrawal responses to heat stimulation in the plantar test showed similar results to the ones observed for the pinprick. Denervated paws did not respond to the hot stimuli on the lateral region until 45 dpi and at this time point group LM + MP.NGF/NT3 showed a shorter latency compared to control, FN and FN + MP.BDNF groups (*p* < 0.01; Figure 4D), indicating that more sensory fibers arrived to the plantar skin of the paw. However, all groups showed similar latency at 60 and 75 dpi.

To further corroborate the functional results, skin reinnervation of the lateral paw pads was analyzed by immunohistochemistry. In all the rats PGP immunolabeling showed regenerated nerve fibers that surrounded the SG tubules, reached the subepidermal nerve plexus, and extended to intraepidermal terminals and Meissner corpuscles at the papillae (Figure 4E–J). Group LM + MP.NGF/NT3 had significantly higher number of IENF than all the other groups (Figure 4K). Similarly, the number of reinnervated SGs was highest in group LM + MP.NGF/NT3, although only significantly from groups COL and LM (Figure 4L).

Taken together, these results indicate that the combination of LM and NGF/NT3 enhances sensory axons regeneration and skin reinnervation by populations of sensory neurons that contribute to thermal and mechanical sensibility, and less markedly of sympathetic fibers innervating the SGs.

## 3. Discussion

NTFs and ECM components both have important roles in nerve regeneration after injury, including effects on Schwann cell migration and differentiation, neuronal survival, cell adhesion and axonal growth [10,23]. To corroborate the preferential effect of NGF, NT-3 and LM on sensory neurons and BDNF and FN on motor neurons [15,24,25,26,27], we cultured DRG explants and spinal cord slices. Organotypic cultures are multicellular in vitro models in which neurons and growing neurites share similar differentiation and development patterns with in vivo conditions [28] while they are still in contact with Schwann cells and fibroblasts. In this way, DRG explants have been long used to study axonal growth and regeneration of the sensory nervous system [29,30] as DRG contain the soma of pseudounipolar sensory neurons that project growing neurites outside the ganglion. On the other hand, neurites growing from the ventral areas of the spinal cord arise from motoneurons of the ventral horn instead of other interneurons [31] making this 3D culture an useful model for studying regeneration of motor neurites in vitro [32].

Taking into account that some NTFs show an attractive or repulsive effect depending on the presence of different ECM molecules [33], we performed a screening of possible combinations added to a collagen gel substrate to investigate if the preferential effects of ECM components and NTFs could be synergistically added in DRG and SC postnatal cultures. We observed an increased effect of LM and NGF + NT3 on sensory neurite outgrowth in DRG explants, whereas this combination did not increase motor neurite length. On the other hand, BDNF combined with FN promoted a synergic effect on motor neurite outgrowth with small effects on DRG explants, which can be attributed to the effect on regenerating proprioceptive neurites [14]. The synergistic effect can be exemplified for BDNF, that promotes sensory neurite outgrowth in the presence of LM but not when is added alone.

These proregenerative effects may be mediated by the differential expression of integrin and NTF receptors in regenerating neurons. It has been described that after injury integrin receptors α7β1 and α5β1 are upregulated in both motor and sensory neurons [14,34], whereas high affinity TrkA and TrkC receptors are expressed in sensory neurons and TrKB is mainly expressed in motoneurons and in a low percentage of sensory neurons [35]. It can be hypothesized that the partially selective in vitro effects of NTFs may be enhanced by the presence of certain ECM molecules in the substrate. In fact, it has been reported that the pro-regenerative effects of NGF and NT3 are reduced after blocking the α7 subunit of integrins in sensory neurons [36], and that synergistic actions between integrins and NTF receptors may be attributed to the sustained activation of Src and the downstream signaling Akt intermediate [37]. On the other hand, NTFs may also modulate the expression of different integrin receptor subunits, whose upregulation is low in adult compared to their expression in early postnatal animals [38]. Actually, NGF contributes to enhance ECM signaling by promoting axonal transport and accumulation of β1 integrin in growth cones, thus enhancing neurite outgrowth [39].

Since the optimal developmental window of regeneration varies from E11–E15 in the chick spinal cord to P7 in rats [40] and the effects of different ECM molecules in vitro are lost when switching from postnatal P7 to weaned P21 rats [14], it was necessary to confirm in a model of peripheral nerve injury and regeneration in vivo the effects observed in cultures. The in vivo results demonstrated that introduction of a collagen matrix enriched with LM + MP.NGF/NT3 or FN + MP.BDNF within the tube used for nerve repair in adult rats promoted preferential regeneration of sensory and motor axons respectively. The NTFs were encapsulated in PLGA microspheres as they have been approved by the FDA as a drug delivery system [41] and we have recently demonstrated that they do not interfere with axon regeneration and their slow release over more than 30 days provides more sustained support for nerve regeneration with respect to addition of free NTFs [21].

All the treated groups showed an increased number of retrogradely traced motor and sensory neurons that had regenerated their axons to the site of tracer application distal to the tube, compared to the control COL group. This is a positive indication that the design and the concentrations of encapsulated NTFs and ECM components did not cause detrimental effects such as the candy store effect [42] or neuronal death induced by high concentration of NTFs [43]. However, the preferential effect mediated by LM + MP.NGF/NT3 and FN + MP.BDNF treatments was more modest compared to the in vitro experiments. This comparative reduction may be explained because of the more complex environment present in the regenerating nerve. After nerve injury, ECM components are synthesized and secreted by non-neuronal cells such as Schwann cells and fibroblasts [44], whereas NTFs are expressed by both neuronal and non-neuronal cells [16,45,46]. Furthermore, non-neuronal cells involved in Wallerian degeneration also express integrins and NTF receptors. Therefore, the in vivo implant of a matrix containing ECM components and NTFs in the nerve conduit does not only influence the injured neurons, but also acts on the migrating non-neuronal cells inside the intratubular matrix. Hence, the activation of non-neuronal cells would contribute with proregenerative non-specific cues and decrease the effects of the selective factors introduced in the exogenous matrix. On the other hand, although we still found in vivo a significant preferential regeneration of sensory neurons in animals treated with LM + MP.NGF/NT3 and of motor neurons in animals treated with FN + MP.BDNF, the differences in the amount of regenerated neurons were reduced from the short to the long term study. We can discard that this could be related to an inefficient supply of NTFs as we have previously shown that NTF encapsulation in MPs improved regeneration of both motor and sensory axons, and that PLGA MPs are able to sustain release of these NTFs longer than a month [21]. The most plausible explanation for the reduced effect observed would be that most axons had passed the site of tracer application at 75 dpi, and thus we were not able to detect differences that occurred at earlier time.

Although retrolabeling of regenerating neurons is a useful technique to assess the differential effect of local treatments, functional restitution is the most important outcome after nerve injury [47]. Thus, in the long term in vivo study we evaluated functional recovery of both motor and sensory targets. Electrophysiological results demonstrated that muscle reinnervation started earlier and achieved higher levels in the FN + MP.BDNF group than in all the other groups. An increased regeneration rate improves muscle reinnervation, particularly of distal muscles in the limb, as shown in the foot muscles in this study, reducing the detrimental consequences of chronic denervation [48]. On the other hand, treatment with LM + MP.NGF/NT3 showed earlier sensory responses to both mechanical and thermal stimuli, confirming the results seen in the retrotracer study. We also demonstrated an increased number of sensory axons reinnervating the epidermis and of sympathetic axons reinnervating the SGs in the skin in the LM + MP.NGF/NT3 group. This parallel effect could be explained by the proregenerative role of NGF on sensory as well as on sympathetic neurons [49].

## 4. Materials and Methods

### 4.1. Ethics Statement

In vitro (procedure #1963M) and in vivo (procedure #1162MM) experimental procedures were approved in 30 June 2015 and in 29 May 2015 respectively by the animal and human experimentation ethics committee (CEEAH) of the Universitat Autonoma de Barcelona in accordance with the European Communities Council Directive 2010/63/EU.

### 4.2. In Vitro Study on Organotypic Cultures

Organotypic cultures were prepared as previously described in detail [32]. Briefly, a 3 mg/mL collagen solution was prepared by mixing rat tail collagen type I (#354236, Corning, Wiesbaden, Germany) with PBS (D8537, Sigma, Tres Cantos, Spain) and sodium bicarbonate at 0.3 mg/mL, and diluting 1:10 with basal Eagle’s medium (10×, Gibco, Grand Island, NY, USA). NTF enriched substrates were prepared by adding BDNF at 50 ng/mL (Peprotech, London, UK) or NGF and NT3 (Peprotech) at 25 ng/mL each, and fibronectin (BD Bioscences, Vienna, Austria) or laminin type I (Sigma) to a 20% final volume. Single 30 µL drops of the prepared matrices were deposited on poly-d-lysine (Sigma) coated coverslips, which were placed in Petri dishes or 24-well multidishes (Iwaki, Asahi Technoglass, Chiba, Japan), and kept in the incubator at 37 °C and 5% CO_2_ for two hours to induce collagen gel formation. Collagen gel was mixed with PBS (COL) and used as control. Collagen gels were also combined with NGF/NT3 (COL + NGF/NT3) or BDNF (COL + BDNF). Similarly, laminin and fibronectin-enriched gels were combined with PBS (LM and FN, respectively), NGF + NT3 (LM + NGF/NT3 and FN + NGF/NT3, respectively) or BDNF (LM + BDNF and FN + BDNF, respectively) (see Table 1).

The lumbar spinal cord (SC, *n* = 6–8/group) and dorsal root ganglia (DRG, *n* = 7–8/group) were harvested from 7-day-old Sprague-Dawley rats, placed in cold Gey’s balanced salt solution (Sigma) enriched with 6 mg/mL glucose and cleaned. SC 350 µm thick slices and DRG explants were placed on gelled collagen droplets, prepared as indicated above, and covered with a second 30 µL drop. The embedded samples were placed in the incubator for 45 min before adding Neurobasal medium (NB, Life Technologies, Carlsbad, CA, USA), supplemented with B27 (Life Technologies), glutamine and penicillin/streptomycin (Sigma).

SC slices were cultured for 4 days, and DRG explants for 2 days. Then, cultures were fixed with 4% paraformaldehyde in PBS for 30 min, and incubated for 48 h with primary antibody mouse RT97 (1:200, Developmental Studies Hybridoma Bank, Iowa City, IA, USA) at 4 °C. After washes, the sections were incubated with secondary antibody AF594 conjugated donkey anti-mouse (1:200, Life Technologies) overnight at 4 °C. For DRG and SC visualization, samples were mounted on slides using Mowiol with DAPI (100 ng/mL, Sigma) for nuclear staining. Olympus BX51 fluorescence microscope (Olympus, Hamburg, Germany) attached to a DP73 camera was used to obtain images of different areas using cellSens Entry software (version 1.12, Olympus), different parts of each sample were merged using Adobe Photoshop CS3 (Adobe System, San Jose, CA, USA).

To analyze the length of neurites, ImageJ software (NIH, available on: http://rsb.info.nih.gov/ij/) resolution parameters were fixed and the three longest neurites were followed from the ventral horn (spinal cord) or ganglion boundary (DRG) to their ending projections. Whole culture images of the DRG and the ventral horn of the SC were analyzed with the Neurite-J plug-in [50] for ImageJ software, and the number of neurites grown at different distances from the explant was compared between sets of cultures. To facilitate the visualization of differences between groups, the area under the curve of each group was converted to a bar plot.

### 4.3. In Vivo Study of Peripheral Nerve Regeneration

Female Sprague-Dawley rats weighing between 250–300 g were used. Animals had ad libitum access to food and water and were kept under a standard light-dark cycle of 12:12 h. All efforts were made to minimize pain and animal distress during surgery.

Rats were anaesthetized with ketamine/xylacine (90/10 mg/kg i.p.), the sciatic nerve was exposed at the midthigh and sectioned 90 mm from the tip of the third toe, and a nerve portion resected. A silicone tube was then sutured with 10-0 monofilament sutures to each nerve stump leaving a 6 mm gap between both nerve ends for the short term study or 8 mm gap for the long term study. Animals were kept for 20 days post-injury (dpi) (short term) or 75 dpi (long term) to allow axonal regeneration before testing.

Each of the three NTFs (NGF, NT3, and BDNF) were encapsulated in microspheres as previously described [51] and added to a collagen solution to reach a final concentration of 2 µg/mL for BDNF and 1 µg/mL for NGF and NT3 respectively. Each preparation of NTF was then added to ECM solutions prepared as for the cultures, i.e., collagen at 3 mg/mL, collagen supplemented with laminin 20% (*v*/*v*), and collagen supplemented with fibronectin 20% (*v*/*v*). Silicone tubes 8 or 10 mm long with an internal diameter of 2 mm were filled with one of the mixtures containing different combinations of ECM substrates and encapsulated NTF. In order to promote fibril alignment, the collagen solution was left to gel vertically for 12 h before surgery [52]. Therefore, there were 7 experimental groups for short and 5 for long term experiments (*n* = 6 per group, see Table 1).

### 4.4. Retrograde Labeling and Neuronal Counting

To quantify motor and sensory regenerated neurons at short term (20 dpi), rats were anaesthetized with ketamine/xylacine and the sciatic nerve was exposed and transected 8 mm distal to the distal end of the silicone tube to apply Fluorogold (FG; 5%; Fluorochrome Inc., Denver, CO, USA) as a retrotracer [53]. Briefly, 5 µL of FG were applied to the end of the nerve for 1 h, then the retrotracer was washed with saline to remove any residues of the tracer and the wound sutured in planes. Similarly, for the long term study (75 dpi) FG was also applied to the tibial nerve at the ankle level. After tracer application, the rats were allowed to survive for 7 days, then, they were deeply anesthetized and transcardially perfused with 4% paraformaldehyde in PBS. The lumbar segment (L3–L6) of the SC and the L4 and L5 DRG were removed, postfixed at 4 °C for 1 h and transferred to 30% sucrose in PBS. Samples were cut in a cryostat longitudinally in 40 and 20 µm thick sections respectively, mounted s severed he tube on slides, heated at 35 °C for 1 h and stored at −20 °C in the dark. DRG and SC sections were observed with an Olympus BX51 fluorescence microscope and the fractionator principle [54] was used to quantify the number of labeled neurons.

### 4.5. Assessment of Muscle Reinnervation

Functional reinnervation of target muscles was assessed at 7, 30, 45, 60 and 75 dpi by means of nerve conduction tests. Animals were anesthetized with ketamine/xylacine and the sciatic nerve was stimulated by transcutaneous electrodes placed at the sciatic notch. The amplitudes (M wave) of the compound muscle action potentials (CMAP) of tibialis anterior (TA) and plantar interossei muscles (PL) were recorded (mV) after placing monopolar needle electrodes in the muscle bellies and the reference in the fourth toe [55]. Values of the contralateral intact limb were used as control. During the tests, the rat body temperature was maintained by means of a thermostated warming flat coil.

Animals were anesthetized with ketamine/xylacine and the sciatic nerve was stimulated by transcutaneous electrodes placed at the sciatic notch.

### 4.6. Assessment of Skin Nociceptive Reinnervation

The progression of nociceptive reinnervation of the hind paw was assessed by means of the pinprick test and thermal sensitivity at 7, 30, 45, 60 and 75 dpi. For the pinprick test, animals were gently kept in a cloth with the sole of the injured paw facing upward, and the skin was stimulated with a needle progressively from proximal to distal at specific sites of the lateral side of the hind paw plantar surface [56]. Fast withdrawal of the hindpaw after stimulation was identified as a clear pain reaction and thus a sign of functional skin reinnervation. The mean number of positive responses in every tested area was calculated per group at each day of testing.

Thermal sensitivity was evaluated using a Plantar test algesimeter (Ugo Basile, Comerio, Italy) [57]. Rats were individually placed in Plexiglas cubicles (20 × 20 × 14 (h) cm) with an elevated Plexiglas floor in a room at constant temperature (24 ± 0.5 °C). The beam of a low intensity lamp (40 mW/cm^2^) was pointed to the lateral part in the hind paw plantar surface with a heating rate of 1 °C/s to elicit activation of unmyelinated C fibers. A cutoff time of exposure to limit possible tissue damage was set at 20 s. The latency (in seconds) of hindpaw withdrawal from the thermal stimulus was recorded as the mean of 3 tests per paw. A 5-min resting period was set between each trial.

### 4.7. Evaluation of Skin and Sweat Gland Reinnervation

For assessing skin reinnervation, plantar pads corresponding to the lateral side were removed at the end of the functional follow-up. Cryotome sections 60 µm thick were processed for immunolabeling against protein gene product (PGP) 9.5 (rabbit; 1:1000; UltraClone, Cambridge, UK), a pan-neuronal marker. Secondary antibodies were conjugated to Cy3. Sections were observed with an Olympus BX51 fluorescence microscope to visualize immunoreactive nerve fibers that had reinnervated the epidermis and the sweat glands (SG). For analysis, images of three sections of each sample were collected with an Olympus DP73 digital camera, the number of intraepidermal nerve fibers (IENFs) was counted in a 1-mm-long segment of the footpad epidermis and the number of reinnervated SGs in the whole pad was also quantified [58].

### 4.8. Data Analysis

Data are presented as mean ± SEM. Results were statistically analyzed using GraphPad Prism (version 6.01, GraphPad Software, San Diego, CA, USA). One- and two-way ANOVA followed by Bonferroni’s post hoc test for comparison between groups were used. Statistical significance was considered when *p*-value was < 0.05.

## 5. Conclusions

In conclusion, this study demonstrates that the interaction between FN + BDNF and between LM + NGF/NT3 has synergistic effects to preferentially enhance motor and sensory axon regeneration, respectively, in vitro. Furthermore, these effects are maintained in vivo in adult animals as motor and sensory axonal regeneration and functional recovery was enhanced after treating nerve injuries with a nerve conduit prefilled with the same combinations of NTFs and ECM components.

## Figures and Tables

**Table 1:** Experimental conditions evaluated in the in vitro and in vivo studies.

Group	Abbreviation	N	Description
**In Vitro Condition**			
Collagen	COL	8	Collagen type I (3 mg/mL) gel
Collagen + NGF/NT3	COL + NGF/NT3	7	Collagen type I (3 mg/mL) gel supplemented with NGF and NT3 (25 + 25 ng/mL)
Collagen + BDNF	COL + BDNF	6	Collagen type I (3 mg/mL) gel supplemented with BDNF (50 ng/mL)
Laminin	LM	8	Collagen type I (3 mg/mL) gel containing 20% laminin type I
Laminin + NGF/NT3	LM + NGF/NT3	7	Collagen type I (3 mg/mL) gel containing 20% laminin type I and NGF + NT3 (25 + 25 ng/mL)
Laminin + BDNF	LM + BDNF	7	Collagen type I (3 mg/mL) gel containing 20% laminin type I and BDNF (50 ng/mL)
Fibronectin	FN	6	Collagen type I (3 mg/mL) gel containing 20% fibronectin
Fibronectin + NGF/NT-3	FN + NGF/NT3	6	Collagen type I (3 mg/mL) gel containing 20% fibronectin and NGF + NT3 (25 + 25 ng/mL)
Fibronectin + BDNF	FN + BDNF	7	Collagen type I (3 mg/mL) gel containing 20% fibronectin and BDNF (50 ng/mL)
**In Vivo Condition**			
Collagen	COL	6	Collagen type I (3 mg/mL) gel
Laminin	LM	6	Collagen type I (3 mg/mL) gel containing 20% laminin type I
Collagen + NGF/NT3 *	MP.NGF/NT3	6	Collagen type I (3 mg/mL) gel containing NGF + NT3 (1 + 1 µg/mL) encapsulated in PLGA microspheres
Laminin + NGF/NT3	LM + MP.NGF/NT3	6	Collagen type I (3 mg/mL) gel containing 20% laminin type I and NGF + NT3 (1 + 1 µg/mL) encapsulated in PLGA microspheres
Fibronectin	FN	6	Collagen type I (3 mg/mL) gel containing 20% fibronectin
Collagen + BDNF *	MP.BDNF	6	Collagen type I (3 mg/mL) gel containing 2 µg/mL of BDNF encapsulated in PLGA microspheres
Fibronectin + BDNF	FN + MP.BDNF	6	Collagen type I (3 mg/mL) gel containing 20% fibronectin and 2 µg/ml of BDNF encapsulated in PLGA microspheres

* Only in the short term study.
